# Green Technology for Baru Oil Extraction Using Solar Energy: Assessing Extraction Efficiency and Process Parameters

**DOI:** 10.1021/acsomega.4c05972

**Published:** 2024-10-18

**Authors:** Lucas Rodrigo Custódio, Caroline Santos Silva, Sandra Cristina Dantas, Kássia Graciele Santos

**Affiliations:** Departamento de Engenharia Química Avenida Randolfo Borges Júnior 1400, Universidade Federal do Triângulo Mineiro, Univerdecidade, Uberaba - MG, CEP, Uberaba 38064-200, Brazil

## Abstract

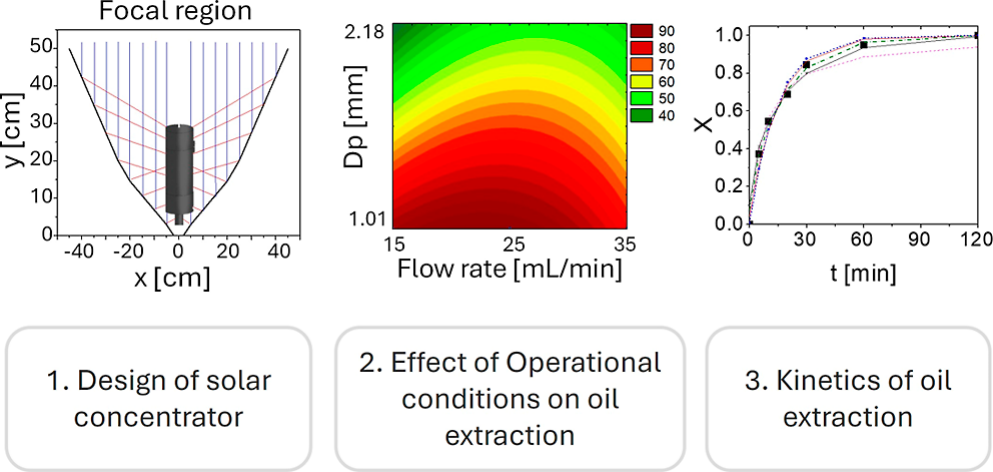

This study explores the application of solar concentrator technology for Baru oil extraction, a crucial resource in various industries. Through systematic experimental investigations, the effects of particle size and solvent flow rate on extraction efficiency were evaluated. Results demonstrate that smaller particle sizes (1.01 mm) significantly enhance extraction efficiency, achieving yields up to 89.25%, compared to larger particles (2.18 mm) with yields averaging 54.32% (±3.2%). Additionally, the solvent flow rate of 25 mL/min maximized extraction efficiency. These findings highlight the critical influence of particle size and solvent flow rate on extraction performance and underline the potential of solar-assisted leaching as a sustainable and environmentally friendly alternative to conventional extraction methods. The research provides a novel contribution to the field by advancing sustainable oil extraction practices and offering a viable solution for small-scale producers to increase the value of their agricultural products.

## 1 Introduction

Vegetable oils are essential commodities across many industries, providing crucial raw materials for food, pharmaceuticals, cosmetics, and biofuels.^1−3^ According to the literature,^4,5^ the health advantages associated with the attributes of diverse fatty acids and other constituents present in vegetable oils have long been acknowledged. Brazil has substantial potential to emerge as a prominent global participant in the investigation, generation, and commerce of vegetable oils and fats.^6^ There is growing attention from the Brazilian government and society toward conserving the Cerrado biome, with initiatives supporting small-scale family production and sustainable extractivism. The Baru tree, a key species of the Cerrado, is threatened with extinction, particularly in states like Mato Grosso, Goiás, Minas Gerais, and the Federal District. Baru almond oil has garnered international recognition for its therapeutic and cosmetic properties,^7^ such as antirheumatic, sudorific;^8^ antioxidant,^9^ tonic, antiaging, wound-healing, cholesterol control, and weight loss facilitation effects.

The high lipid content in Baru nuts results in their oil being primarily obtained through mechanical pressing. According to Borges et al.,^10^ the Baru oil presents a high content of unsaturated fatty acids, particularly oleic (45.83%) and linoleic acid (28.93%), which together account for more than 74% of the total fatty acid composition. These unsaturated fatty acids are known for their beneficial health properties, including anti-inflammatory and cardioprotective effects. Among the saturated fatty acids, palmitic acid (6.37%) and stearic acid (5.28%) are the most abundant, followed by minor quantities of arachidic (1.38%), behenic (3.90%), and lignoceric acid (4.79%).

Crude oils can be extracted using methods like cold pressing or organic solvent leaching.^11,12^ However, conventional oil extraction methods, widely employed in the industry, face numerous challenges, including high energy consumption, extensive use of chemical solvents, and environmental pollution.^13^

Solvent heating in the leaching process is one of the most energy-consuming steps, making the process costly. To address the challenges, researchers have been exploring alternative approaches that utilize renewable energy sources and minimize environmental impact. Using solar energy for heating the extracting solvent emerges as a sustainable and environmentally clean solution that can economically enable small-scale oil production processes due to its abundance, sustainability, and zero emissions.^14^

While previous research has predominantly focused on hydrodistillation methods (Table S1) using solar energy for essential oil extraction, there has been limited exploration of solar-assisted solvent extraction techniques, particularly in fixed-bed systems for oil-resin extraction. This study seeks to introduce an innovative approach that utilizes solar concentrator technology for oil-resin extraction from Baru nuts. The application of solar concentrator technology specifically for this purpose is scarcely documented in the literature, suggesting a potential research gap that our study aims to address. By exploring the feasibility of a low-cost, easy-to-make solar concentrator in oil extraction processes, this research offers a novel perspective and potential advancements in efficiency and environmental sustainability.

The objective of this study was to evaluate the efficiency of solar-assisted leaching for extracting oil from Baru nuts, comparing it with conventional Soxhlet extraction. By analyzing the effects of solvent flow rate and particle size on the extraction efficiency, the study aimed to optimize the parameters for maximum oil yield while considering the impact of temperature variations due to solar radiation. The research also sought to identify the most suitable kinetic model to describe the extraction process and address challenges posed by environmental factors such as cloud cover and wind, which affect the consistency of solar-assisted extraction. Thus, the significance of this research offers a feasible and cost-effective solution tailored to the needs of small producers.

## 2 Material and Methods

The experiments were carried out at the Federal University of Triângulo Mineiro, located in the city of Uberaba, state of Minas Gerais, Brazil, its geographical position is 19° 45′ 27″ south latitude and 47° 55′ 36″ west longitude, which has an average solar radiation through the year of 829 W/m^2^. In the periods between 10:00 AM and 15:00 PM, schedules of higher solar incidence rates, on days of low cloudiness.

### 2.1 Sample Preparation and Characterization

The Baru nut samples were commercially sourced and underwent a standardized preparation process. Initially, the endocarp was cracked open and the shells were removed. Subsequently, the nut samples underwent drying in an oven at 100 °C for 24 h to reduce their water content. Grinding is vital in breaking down seed tissues, enhancing oil extraction by augmenting the surface area for solvent interaction. This enhances solvent diffusion, especially at elevated temperatures approaching the boiling point of the solvent, while also decreasing material moisture.

So, the material was crushed and sieved, employing opening diameters of 2.36, 2.00, 1.18, and 0.84 mm. For the extraction process, the material was divided into two sets of samples, with average particle sizes of 1.01 and 2.18 mm.

The bulk density of the material used in the extraction corresponds to the ratio between the mass of the Baru nut (120 g) and the volume of the packed bed used in the extraction (290 cm^3^), resulting in a bulk density of 0.414 g/cm^3^.

The conventional Soxhlet method was employed to estimate the percentage of oil present in the Baru nuts. For the experiment, a particle size of 1.09 mm was used, with ethanol (96.3%) as the solvent. Soxhlet tests were conducted in duplicate, following the methodology proposed by literature.^15−19^ A sample mass of 15 g is placed inside a cartridge, which is then inserted into the Soxhlet extractor, and subsequently submerged in the solvent. About 150 mL of solvent is added, and water circulation is initiated to condense the solvent and prevent evaporation loss. After a 4 h extraction, the mixture was heated until all solvent had evaporated. Following cooling, the flask containing the oil and the sample particles was taken to the oven. The extracted oil content was calculated by the ratio of mass loss in extraction to the initial sample mass.

### 2.2 Experimental Unit of Extraction

The equipment developed for the extraction tests and the designed solar concentrator are described in this topic.

Initially, the design of the solar concentrator was carried out, which involved defining the geometric characteristics of the prototype. Thus, it was necessary to calculate the concentration point of solar radiation, namely its focus. According to the literature,^20^ by considering the principles of reflection and trigonometry, it is possible to determine the focal points of any type of curvature. Therefore, to calculate the concentration point of solar radiation, the methodology developed by this author was used, which consists of the following steps:

- Plot the mathematical function *f*(*x*_p_) representing the curve responsible for generating the collector’s structure on a Cartesian plane.
- Given any point *P* with coordinates (*x*_p_, *y*_p_), it is necessary to determine the tangent line to this point, denoted as line *t.* The slope of this line is the derivative of the generating function *f*′(*x*_p_) and is represented in eq 1.^20^ Additionally, it is essential to determine the line perpendicular to this at point *P*, called the normal line or line *n*, represented by eq 2:^20^

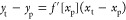
1
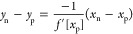
2

- After determining the lines *t* and *n*, the solar incidence ray is plotted, represented by line *i*, and given by^20^

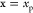
3

- The angle between the incident ray (line *i*) and the normal (line *n*) is the same angle between the normal and the reflected ray, denoted as θ^20^

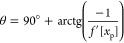
4

- Thus, being the same angle, to determine the reflection of the incident ray, eq 5 is used,^20^ represented by line *r*

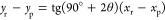
5

- Finally, the focal point *F* of interest will lie on the *y* axis, where the line *r* intercepts it, that is, *F*(0,*y*_F_), where the ordinate *y*_F_ is given by eq 6:^20^

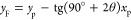
6

**Figure 1:**
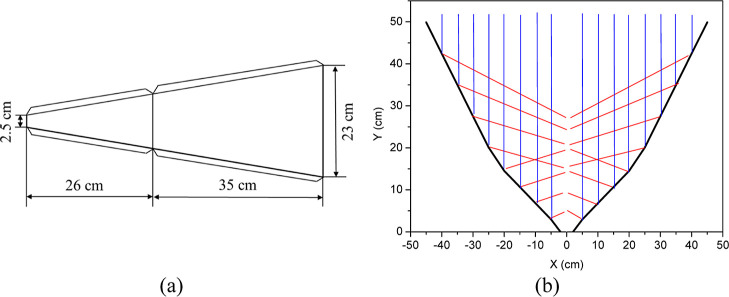
TSBSC: (a) dimensions of the sides of the solar concentrator plates; (b) cross-sectional profile of the concentrator.

The solar concentrator consisted of 12 galvanized steel plates fixed together by their edges. A schematic in Figure 1a illustrates the dimensions of the concentrator’s sides when assembled to form a quasi-parabolic shape, also called as twelve-sided biangular solar concentrator (TSBSC).^21,22^ The inner surface of the concentrator is covered with a metallic adhesive film to enhance solar radiation reflection.

In contrast to a conventional parabolic solar concentrator, characterized by a single focal point, the TSBSC offers a large focal region. Notably, the concentrator’s internal angle ensures an expanded upper aperture to capture solar rays effectively, concentrating them within a linear area spanning 25 cm of the focal region. In Figure 1b, the schematic illustrates the incidence (blue) and reflection (red) of solar rays, showcasing the concentrator’s optimal functionality, calculated from eqs 1 to 4.

**Figure 2:**
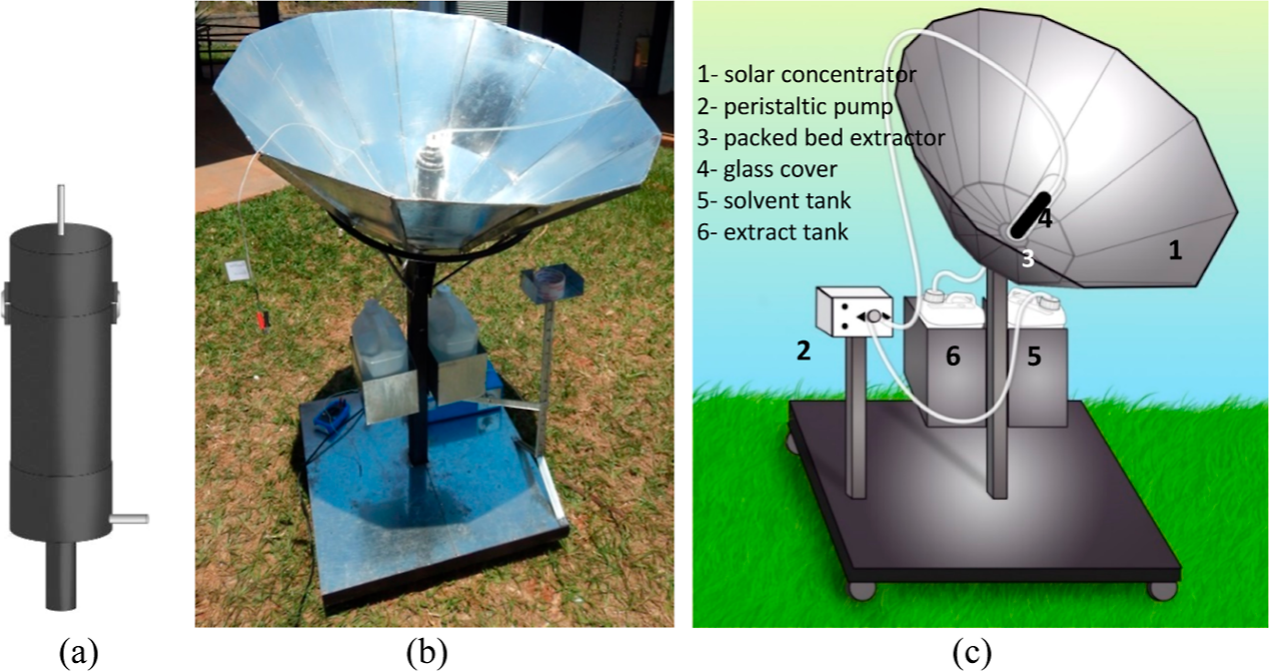
Experimental unit of solid–liquid extraction: (a) absorbent bed; (b) experimental apparatus photo; (c) schematic representation of the experimental unit.

Figure 2a provides a detailed illustration of the extraction bed, showcasing its composition and design. The bed comprises a cylindrical metal tube crafted from copper, measuring 16 cm in height and 5 cm in diameter. Notably, copper’s excellent thermal conductivity makes it an ideal material for heat transfer applications. To enhance heat absorption, the outer surface of the bed is coated in black paint. Moreover, to minimize heat loss and optimize temperature retention, the bed is encased in a glass cover, as depicted in Figure 2b. Functioning akin to a greenhouse, the glass cover permits the transmission of infrared rays while trapping heat within the enclosure.

Figure 2c presents a comprehensive depiction of the experimental unit’s structure, elucidating its configuration and functionality. In addition to the extraction bed and concentrator, this unit includes two storage reservoirs. One reservoir holds the solvent, while the other receives the extract through a duct positioned at the base of the bed. To facilitate solvent distribution, a peristaltic pump (5 W) delivers the solvent feed to the bed’s surface. Supporting the concentrator and auxiliary components, a metal base featuring an east–west sloping axis enables adjustment of the concentrator according to the sun’s position.

### 2.3 Solar-Assisted Leaching

**Figure 3:**
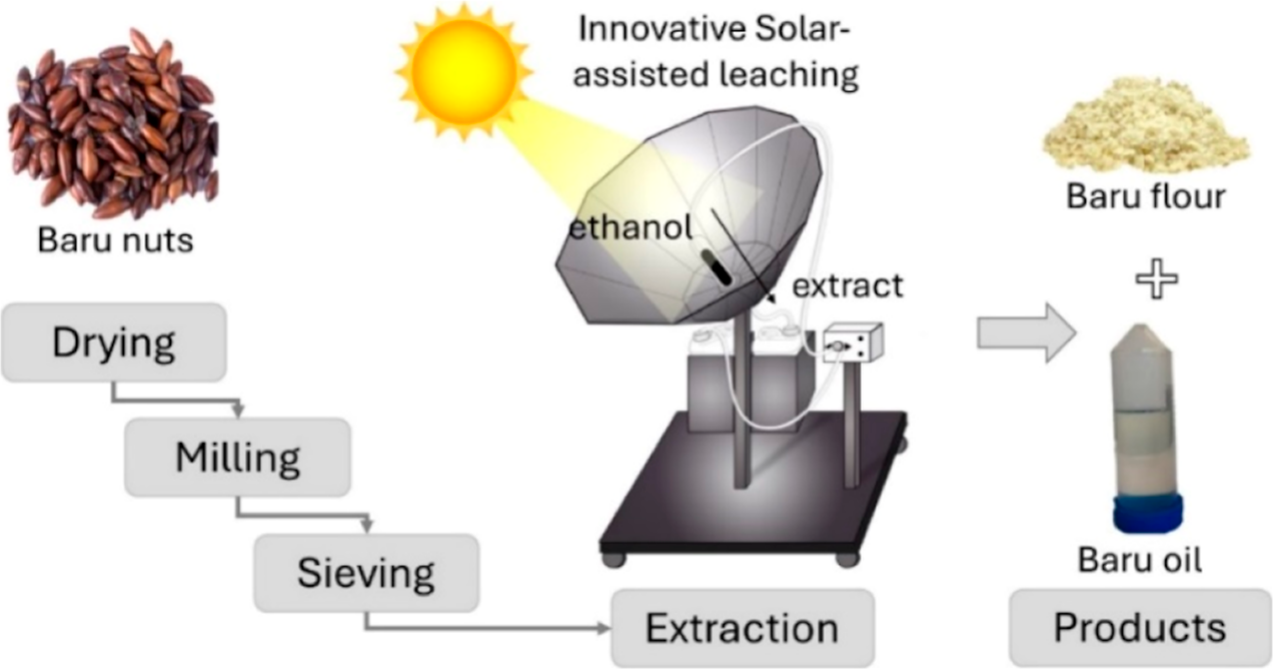
Graphical representation of the solar-assisted leaching process for nut preparation and product extraction.

Figure 3 presents a graphical representation of the solar-assisted leaching process, detailing each step from nut preparation to the final products. In the solvent extraction process, flow rate is commonly manipulated to establish desired operating conditions. Increasing the flow rate typically enhances extraction up to a certain point by increasing convective mass transfer coefficients. However, this increase also reduces solvent residence time, thus diminishing fluid-particle contact time, essential for diffusion, both from the solvent into the pores and from the micelle into the solution. In the case of extraction in the solar concentrator, flow rate variation also influences the extraction fluid temperature, where longer residence times lead to higher operating temperatures. It is ideal for the process to operate near the solvent’s boiling temperature, as at this temperature, there is a decrease in oil viscosity and an increase in its solubility, allowing ethanol to interact with the oil and transport it out of the cell.

The experiments were carried out on different days; however, to ensure smooth operation, the selected days presented favorable environmental conditions, namely clear skies and ambient temperatures above 25 °C.

In this study, the analyzed factors were particle size (sample A with 1.01 mm and sample B with 2.18 mm) and solvent feed rate (15, 25, and 35 mL/min). The solvent used was ethanol (96.3%), fed at the determined flow rate through a peristaltic pump. Each experiment had an extraction time of 2 h with continuous feeding. Throughout the experiment, the temperature was measured at the packed bed wall, inner and outer glass walls, air trapped inside the glass, and the extractor’s exiting extract, using type K thermocouples.

At the end of each run, the cake was taken to the oven for 24 h. The extracted oil mass was calculated by the difference between the initial sample mass and the mass of the dry cake. The extraction yield (η) was calculated as the ratio of the oil extracted in the solar energy experiment to the oil extracted in the Soxhlet extraction. Table 1 presents a specification list of all equipment used in the experiments in this study.

**Table 1:** Equipment Specifications Used in This Work

equipment	specification
digital multimeter associated with K-thermocouple	Minipa, ET-2042F, –40 to 1000 °C
peristaltic pump	FA AMBIENTAL (FE) 12 V; 5 W
Willye type mill	R-TE-650/1—Tecnal −500 W
Soxhlet fat extractor	Marconi—MA487/6/250, 6 tests
electromagnetic sieve shaker	Bertel, AGT.P-220 V – 360 W

### 2.4 Extraction Kinetics

The kinetic study was performed for Baru nut particles of 1 mm, at the best condition of solvent flow rate. For the solar concentrator extraction kinetics test, six samples of 50 g of the Baru nuts. Analyses were conducted at collection times of 5, 10, 20, 30, 60, and 120 min. At the end of each collection, the cake was sent to the oven at 100 °C for 24 h. After this period, the sample was weighed to determine the amount of retained oil at each time.

In this study, some kinetic models were selected for parameter adjustment to characterize the extraction kinetics of Baru oil: the Esquivel model,^23^ diffusive model,^24^ Crank model,^25^ simple single plate (SSP) model^26^ and logistic model (LM).^27^ These models are usually used to describe the oil extraction kinetics of many materials (see Table S2 in Supporting Information file). The concordance between the experimental observations and the predicted values was assessed using the coefficient of determination (*R*_adj_^2^) and the root-mean-square deviation (RMSD), as described in eq 7.
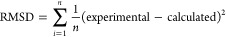
7

## 3 Results and Discussion

The conventional extraction method using the Soxhlet apparatus was employed to estimate the amount of oil initially present in the Baru nuts. Table 2 presents the results of the extraction test conducted in duplicate, from which it was estimated that the Baru nuts contain approximately 34.53% by mass of oil-resin. This value was used as a reference in calculating the extraction yield using the solar-powered extractor. A similar result was obtained by Fetzer et al.^13^ The authors evaluated the extraction of Baru oil from particles with a diameter of 1.0 mm using ethanol as a solvent in a Soxhlet apparatus and achieved an extraction yield of 36.84 wt %.

**Table 2:** Soxhlet Extraction: Initial Mass of Baru Particles (*W*_0_), Cake Mass (*W*_cake_), Oil Mass (*W*_oil_), and Baru Oil Content

*W*_0_ [g]	*W*_cake_ [g]	*W*_oil_ [g]	oil content (%)	mean oil content (%)
15	9.80	5.20	34.63	34.53 ± 0.13
	9.83	5.17	34.45	

In the solar-assisted leaching process, the cool solvent is pumped to the packed bed, and it is heated due the heat transfer with the wall of the bed and the particles. The solar radiation and the flow rate directly affect the process temperature. Without the interference of solar radiation, a low flow rate could cause higher temperatures due to the low residence time in the bed, while high flow rates could cause a cooling of the system. High temperatures are not always desirable for the process, as they may cause solvent evaporation and thermal degradation of the oil. However, it is not possible to control the climatic conditions and consequently variations in the process temperature will occur, which may impact extraction efficiency.

**Figure 4:**
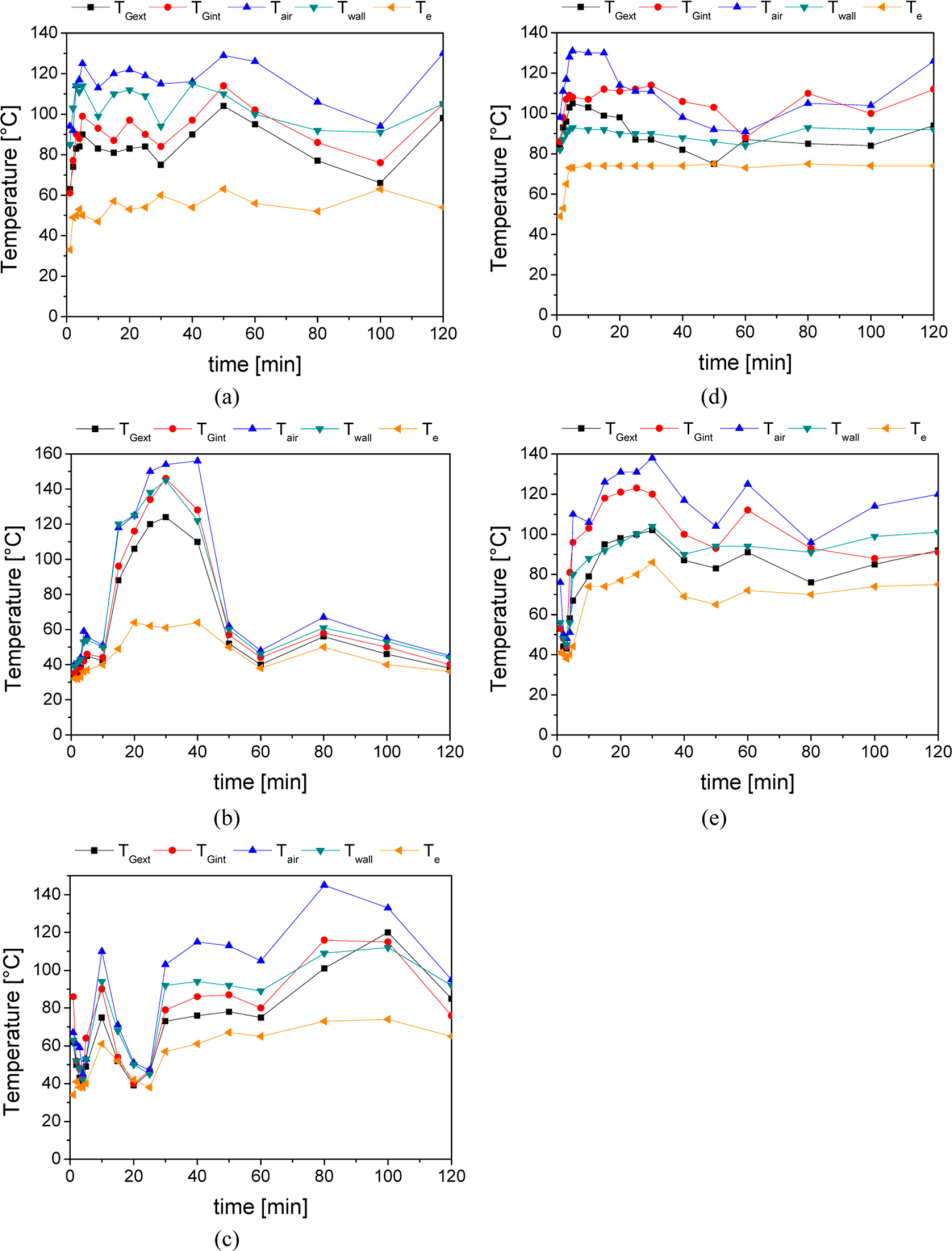
Temperatures variations with the time at: external (*T*_Gext_) and internal (*T*_Gint_) wall of the glass cover, at the bed wall (*T*_wall_), in the air between the glass cover and the bed (*T*_air_), and at the outlet of packed bed (*T*_e_): (a) *Q* = 15 mL/min and dp = 1.01 mm; (b) *Q* = 25 mL/min and dp = 1.01 mm; (c) *Q* = 35 mL/min and dp = 1.01; (d) *Q* = 25 mL/min and dp = 2.18 mm; (e) *Q* = 35 mL/min and dp = 2.18 mm.

In this way, the temperatures were recorded at important points in the system, allowing a better understanding of the process: at the external (*T*_Gext_) and internal (*T*_Gint_) walls of the glass cover; at the bed wall (*T*_wall_), in the air between the glass cover and the bed (*T*_air_), and at the outlet of packed bed (*T*_e_). These temperatures were recorded along the 2 h of the test and they are displayed at Figure 4. It can be seen that experiments 2 and 3 displayed significant temperature variations attributable to the presence of clouds and wind during the testing period, resulting in diminished solar radiation reception.

The highest temperatures were observed in the air present between the glass cover and the bed wall, reaching 156 °C. This is attributed to the greenhouse effect induced by the glass enclosure. Specifically, the glass cover used is transparent to visible light, with a transmittance of approximately 90%,^28^ allowing solar radiation to pass through and be absorbed by the absorber bed, which subsequently heats up. However, the glass has low transparency to infrared radiation emitted by the heated absorber bed, with a transmittance of around 10% for infrared wavelengths. This property prevents the escape of the generated heat, effectively trapping it inside the concentrator. Consequently, the trapped heat raises the temperature of the air in the space between the cover and the absorber bed, enhancing the overall efficiency of the solar concentrator system.

The second highest temperature was recorded at the bed wall (*T*_wall_) situated at the focal point of the solar concentrator, followed by the internal and external temperatures of the glass cover. As a result, the heat absorbed by the metal bed’s walls is transferred to both the particles and the fluid flowing through the bed, thereby warming the micelle. Consequently, the micelle exhibits the lowest temperatures.

Table 3 presents the days and time of each test, experimental conditions and the responses from solar-assisted leaching, as well as the room temperature and average solar radiation during the experiment, recorded by the Instituto Nacional de Meteorologia at Uberaba, MG, Brazil (INMET).^29^

**Table 3:** Experimental Conditions of Solvent Flow Rate (*F*) and Particle Diameter (Dp) and the Main Responses From Solar-Assisted Leaching: Extracted Oil Content, Extraction Efficiency of Baru Oil (η), Maximum (*T*_Emax_) and Average (*T*_Ea_) Temperature of Extract, Room Temperature (*T*_a_) and Average Solar Irradiation (*I*)

			factors		responses					
run	day	time	*F* (mL/min)(X1)	Dp [mm] (X2)	oil content (%)	η (%)	*T*_Emax_ (°C)	*T*_Ea_ (°C)	*T*a^b^ (°C)	*I*^b^(W/m^2^)
A	12/14/2017	11:00–13:00	15 (−1)	1.01 (−1)	28.61	82.83	63	55.8	25.8	895.9
B	12/15/2017	14:00–16:00	25 (0)	1.01 (−1)	30.82	89.25	64	46.6	28.5	767.6^a^
C	01/12/2018	14:00–16:00	35 (+1)	1.01 (−1)	25.67	74.31	74	63.4	26.7	607.4^a^
D	11/14/2017	13:00–15:00	25 (0)	2.18 (1)	18.76	54.32	75	73.7	31.9	1042.6
E	11/16/2017	13:00–15:00	35 (+1)	2.18 (1)	17.93	51.90	77	71.7	31.0	1038.6

a Strong winds and large variations in solar radiation due to cloudiness.

b Data from INMET.^29^

The presence of clouds impacted the average solar radiation received during the experiments 2 and 3, hindering the ability to establish a direct relationship between the working flow rates and the average temperatures obtained in the tests. In solvent extraction processes, the manipulation of flow rate is a common practice to optimize operational parameters. Incremental increases in flow rate generally augment extraction efficiency up to a critical threshold, attributed to the elevation in relative velocity between the fluid and the particle, consequently amplifying convective mass transfer coefficients. Nevertheless, elevated flow rates correspondingly diminish solvent residence time, thereby curtailing the duration of fluid-particle interaction crucial for diffusion mechanisms, encompassing both solute penetration into the pores and micelle diffusion into the solution phase.^21^

It is observed at Table 3 that extraction using smaller particles (1.01 mm) resulted in higher extraction efficiency compared to particles with a diameter of 2.18 mm, this greater efficiency can be attributed to the larger contact surface. According to the literature,^30^ it is known that reducing the particle size of the biomass material improves the yield of extracted oil, due to the increase in the surface contact area between the biomass and the solvent. The highest yielding extraction, η = 89.25%, was obtained for a feed rate *F* = 25 mL/min and Dp = 1.01 mm, as observed in a previous works on oil extraction from Jatropha^21^ and pequi.^31^

**Figure 5:**
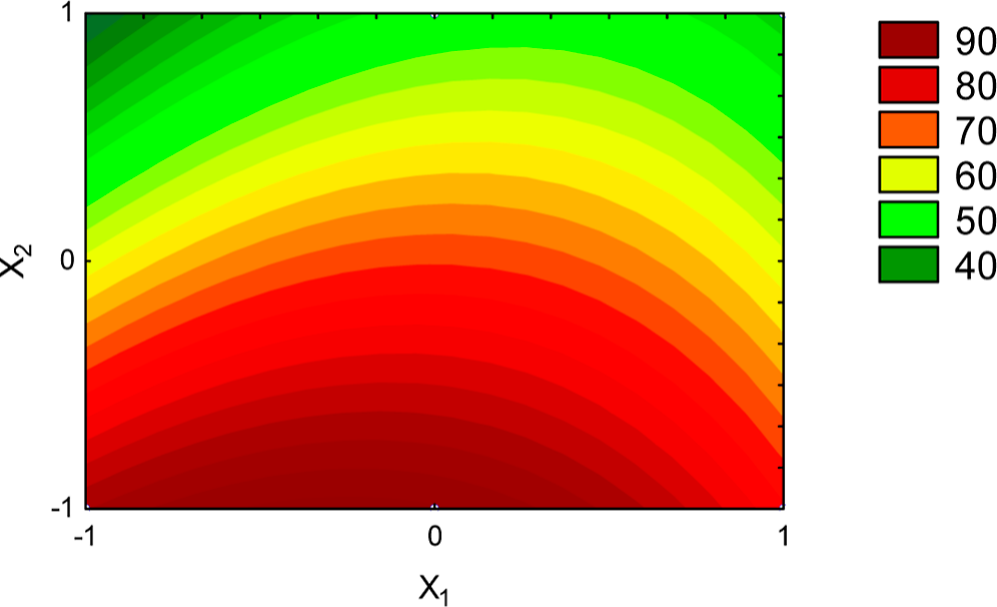
Extraction efficiency of Baru oil (η (%)) as a function of factors (X_1_—solvent flow rate and X_2_—particle diameter).

An analysis of variance (α = 20%) was conducted to assess the effect of factors F and Dp on the extraction efficiency. This high value of p-level was used because the temperature of process also can influence the extraction efficiency, and it cannot be controlled in the solar-assisted leaching. So, the temperature differences are accounted in the error. Table 4 presents the estimated effects of the factors on the extraction efficiency that were considered significant, while Figure 5 shows the contour plot of effect of factors on the extraction efficiency.

**Table 4:** Effect of Main Coded Factors (X1—Solvent Flow Rate and X2—Particle Diameter) on Extraction Efficiency of Baru Oil (η) (*R*_adj_^2^ = 0.98579)

factors	effect	standard error	p-level
mean	65.665	1.000	0.010
X_1_ × X_1_	9.180	1.936	0.132
X_2_	–32.930	2.000	0.039
X_1_ × X_2_	9.520	2.646	0.173

These findings underscore the significant influence of particle diameter on the extraction process. The particle diameter was the main factor that influences the extraction process. The inverse correlation observed between particle diameter and extraction efficiency highlights that smaller particles facilitate more efficient extraction. This phenomenon can be attributed to the fast internal mass transfer facilitated by diffusion within smaller particles. Conversely, larger particles, such as those with a diameter of 2.18 mm, tend to exhibit lower extraction efficiency due to the limitations imposed by high internal mass transfer resistance. Furthermore, the decrease in particle diameter leads to an augmented surface area available for external mass transfer, thereby facilitating oil extraction.^30^

The flow rate of the solvent also plays a critical role in the solid–liquid extraction process, directly affecting its efficiency, showing a significant quadratic effect. As the relative velocity between the solvent and the solid particles increases, so does the convective mass transfer coefficient, leading to improved extraction efficiency. In continuous extraction processes, an increase in solvent flow rate results in a proportional enhancement of oil mass transfer from the solid phase to the solvent phase. However, it is essential to consider the properties of the solvent itself; in this case, ethanol, with a boiling point close to 78 °C, which undergoes heating within the extraction column. Consequently, variations in fluid residence time influence the operating temperature, inversely correlated with the flow rate. However, due to the significant variations in solar radiation that interfered with extraction temperatures, it was not possible to assess the impact of flow rate on the temperatures obtained during the test.

Upon observation, the lowest flow rate (*F* = 15 mL/min) resulted in a longer residence time of the solvent within the bed resulting in high contact time but low solid–liquid relative velocity, which also reduces the convective mass transfer coefficient. Conversely, the highest flow rate (*F* = 35 mL/min) exhibited the highest solvent velocity, which increases the convective mass transfer coefficient, but also resulted in a shorter residence time of the solvent in contact with the particles. Hence, the intermediate flow rate (*F* = 25 mL/min) proved to be more efficient in oil extraction due to its combination of elevated mass transfer coefficients and an intermediate residence time, facilitating the contact between phases. Consequently, both diffusion and external mass transfer processes occurred unhindered, thereby yielding increased extraction efficiency.^21^

Extraction of oil in packed bed columns involves a series of interconnected steps, elucidating the intricate kinetics of the process. Initially, the solid–liquid extraction occurs under transient conditions, encompassing a multitude of physical phenomena. The mass transfer process occurs within three distinct regions: the solid matrix of the particle, the stagnant miscella within the particle pores, and the convective miscella region within the bed voidage. Understanding the dynamics within these regions is crucial for elucidating the extraction mechanism.

**Figure 6:**
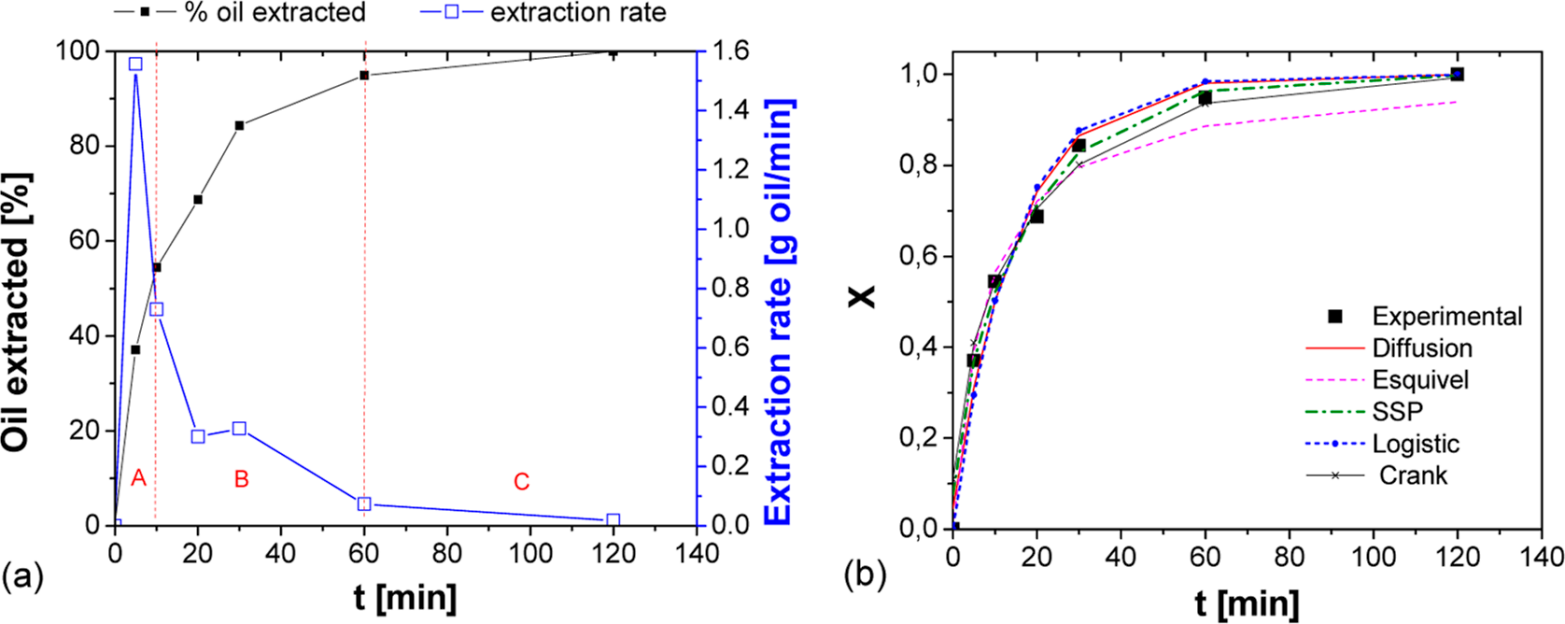
Extraction kinetics of Baru oil in a packed bed: (a) experimental percentage of oil extracted; extraction rate as a function of time and the main stages of extraction (A)—constant rate stage, B—decreasing rate stage; and C—diffusive stage; (b) comparison with simulated kinetic models and experimental data.

The kinetics of oil extraction in packed bed columns unfold through discernible phases as presented in Figure 6a, each illuminating key facets of the process dynamics, where X is the ratio between the oil extracted over time and total initial oil content.

Initially, a rapid escalation in extraction rate is observed (stage A), characterized by a steep ascent in the extraction curve. This is also called the filling stage. The oil extraction in fixed-bed columns is initiated with the column filled solely with solid material. As the solvent is introduced at the top of the bed, it begins to wet the surface of the solid material. Consequently, the pores within the solid material, both between particles (interparticle) and within particles (intraparticle), start to be filled with pure solvent. This action allows the solvent to percolate through the bed, extracting oil from the solid material as it progresses downward. This filling stage exhibits increased complexity compared to when the bed is already fully saturated with solvent. One key reason for this complexity is that during filling, there is a leading edge of solvent (miscella) that gradually moves down the column. As this leading edge progresses, it occupies both the bulk region (between solid particles) and the pore region (within and between particles) of each new section it encounters. This gradual occupation of the bed by the solvent creates an increasing extraction rate over time.^32^

In the final stage (stage C) of the extraction process, the outer layer of solute within the solid matrix becomes exceedingly thin, nearly vanishing. Consequently, the mass transfer primarily relies on diffusion mechanisms. As the concentration gradient between the solid matrix and the solvent diminishes, the rate of diffusion slows down, leading to a gradual decline in the overall extraction rate.^33^

According to Figure 6a, the highest oil extraction rate occurred within the initial 20 min, reaching a maximum of 1.56 g oil/min in this small-scale test. Approximately 95% of the oil was extracted within the first 60 min of operation. Subsequently, during the subsequent 60 min, the extraction rate significantly decreased as the oil to be extracted was located within the particles, hindering mass transfer processes. Therefore, to enhance extraction productivity, experiments lasting for 1 h are recommended, resulting in a 5% reduction in yield but allowing for experiments to be conducted in half the time.

Table 5 presents the adjusted model parameters, alongside the coefficient of determination (*R*^2^) values for model fits and RMSD. Additionally, Figure 6b illustrates the fitting of models with experimental data.

**Table 5:** Parameters of Kinetic Models and Statistical Analysis for Baru Oil Extraction in Packed-Bed Columns

model	parameter	estimated	std. error	p-level	*R*_adj_^2^	RMSD
Esquível^23^	*b*_1_	7.693	0.776	6.10 0 × 10–5	0.9841	1.94 × 10^–3^
Diffusive^24^	*A*	0.955	0.043	3.00 0 × 10–6	0.9846	1.40 × 10^–3^
	*B*_1_	0.065	0.006	1.73 0 × 10^–4^		
Crank^25^	*De* (cm^2^/min)	3.8 0 × 10–5	0.000	0.00	0.9789	1.27 × 10^–3^
SSP^26^	*De* (cm^2^/min)	5.2 0 × 10^–5^	0.000	0.00	0.9899	2.35 × 10^–3^
Logistic^27^	*b*	0.070	0.010	1.03 × 10^–3^	0.9814	2.66 × 10^–3^
	*tm*	–149.390	1.805 × 10^5^	0.994		

The Esquível model^23^ yielded a highly significant parameter estimate (*b*_1_ = 7.693, *p* < 0.001), indicating a strong correlation between the model and the experimental data. The high *R*_adj_^2^ of 0.9841 and low RMSD of 1.94 × 10^–3^ further support the model’s goodness of fit.

The diffusive model^24^ exhibited a significant parameter estimate for parameter *A* (*A* = 0.955, *p* < 0.001), indicating a strong influence of diffusion mechanisms on the extraction process. The *R*_adj_^2^ of 0.9846 and RMSD of 1.40 × 10^–3^ suggest a high level of agreement between the model and the experimental data.

For the Crank model,^25^ the parameter estimate for the diffusion coefficient (De) was significant (De = 3.8 × 10^–5^ cm^2^/min), with a high *R*_adj_^2^ (0.9789) and low RMSD (1.27 × 10^–3^), indicating a good fit of the model to the data. Similarly, the SSP Model^26^ exhibited a significant estimate for De (De = 5.2 × 10^–5^ cm^2^/min), with a high *R*_adj_^2^ (0.9899) and low RMSD (2.35 × 10^–3^), suggesting a strong correlation between the model and the experimental data.

The LM^27^ yielded parameter estimates for *b* (*b* = 0.070, *p* = 0.001) with statistical significance, suggesting its influence on the extraction kinetics. However, the estimated parameter tm (−149.390) lacked statistical significance (*p* = 0.994). Typically, tm represents the time at which the highest mass transfer rate occurs. The insignificant and negative adjusted value of tm raises doubts about the model’s ability to capture this physical phenomenon accurately for Baru oil extraction in fixed-bed columns. Further exploration and alternative modeling approaches are warranted to better characterize the extraction kinetics.

In summary, based on the statistical analysis presented, the Diffusive model^24^ appears to be the most suitable for representing the kinetics of Baru oil extraction in fixed-bed columns, as it exhibited the highest *R*_adj_^2^ value and lowest RMSD among the models evaluated. However, further validation and sensitivity analyses may be warranted to confirm the robustness of the selected model.

One limitation encountered during this investigation was the impact of climatic variations on extraction efficiency. As observed in experiments 2 and 3, the presence of clouds and wind significantly reduced solar radiation reception, resulting in notable temperature fluctuations within the extraction system. These environmental factors hindered the ability to establish a direct correlation between solvent flow rates and process temperatures, thereby affecting the consistency and reproducibility of the extraction outcomes. Additionally, the inability to control ambient temperatures and solar radiation levels underscores the inherent variability associated with solar-assisted extraction methods. Future studies should explore strategies to mitigate these climatic influences, such as integrating thermal storage systems or hybrid energy sources, to ensure a more stable and controlled extraction environment.

The achieved outcome of this study proves highly satisfactory for small installations, positioning the extraction method as nearly entirely sustainable, given the minimal energy expenditure of the pump, clocking in at less than 0.01 kWh. Additionally, solvent feeding can be facilitated through gravity, with the feeding tank positioned above the bed, and flow rate adjustment achieved through mechanisms such as a hospital serum valve. An additional noteworthy aspect pertains to the equipment’s low construction cost, which allows for the use of disposable materials like cardboard.^21^ Moreover, the operational expenses of the process are limited to the commercial value of the solvent, which can further be recovered through solar distillation.

## 4 Conclusions

This investigation demonstrates the viability and effectiveness of solar-assisted leaching for Baru oil extraction, highlighting its potential for sustainable oil production. By harnessing solar energy to elevate solvent temperatures within fixed-bed columns, substantial oil extraction efficiencies were achieved, comparable to conventional methodologies. The oil-resin content of Baru nuts was determined to be approximately 34.53% w/w. An extraction efficiency of 89.25% was achieved with a solvent flow rate of 25 mL/min and a particle diameter of 1.01 mm using the solar-assisted leaching process, compared to the Soxhlet extraction.

Notably, the results indicated that particle diameter exerts a more significant influence on extraction efficiency compared to solvent flow rate. Smaller particle sizes demonstrated heightened extraction efficiencies, emphasizing the importance of particle size optimization in the leaching process.

Statistical analysis of kinetic models highlighted the substantial influence of diffusion mechanisms, with the diffusive model^24^ emerging as the most suitable for elucidating the kinetics of Baru oil extraction.

This study contributes to the advancement of knowledge and practices within the realms of sustainable agriculture and renewable energy. Ultimately, this investigation not only underscores the promise of solar-assisted leaching for sustainable oil production but also holds potential for empowering small-scale producers and advancing broader sustainable development objectives. So, this research paves the way toward a more sustainable and environmentally conscientious future within the agricultural domain.

Despite the promising results, the solar-assisted leaching method has some limitations. A major drawback is the process’s sensitivity to climatic conditions, such as fluctuations in solar radiation and ambient temperature, which can affect the efficiency and reproducibility of the extraction process. Additionally, the reliance on solar energy restricts the method’s applicability in regions with inconsistent sunlight or during periods of low solar intensity.

Future research should explore integrating hybrid systems that combine solar energy with alternative power sources to maintain consistent operation despite solar intensity variations. Developing more efficient solar collectors and designing adaptable, modular systems for varying environmental conditions are also recommended. Incorporating real-time monitoring and feedback mechanisms can enhance process control precision and system reliability. Additionally, performing cost-benefit analyses and life cycle assessments will be essential for evaluating the method’s economic feasibility and environmental impact.

## Supporting Information Available

Two tables with literature review on leaching assisted by solar energy and kinetic models of oil extraction (PDF)
