# Supplementary material for: Green Technology for Baru Oil Extraction Using Solar Energy: Assessing Extraction Efficiency and Process Parameters
Source: ACS Omega. 2024 Oct 18;9(43):43668–77. doi: 10.1021/acsomega.4c05972 (PMC11525507; doi:10.1021/acsomega.4c05972)
Supplement: Supplementary file 1 — ao4c05972_si_001.pdf [file ao4c05972_si_001.pdf]

Supporting Information

Green Technology for Baru Oil Extraction Using Solar Energy: Assessing Extraction Efficiency and Process Parameters

Lucas Rodrigo Custódio[a], Caroline Santos Silva[a], Sandra Cristina Dantas[a], Kássia Graciele dos Santos[a*]

[a] Universidade Federal do Triângulo Mineiro, Departamento de Engenharia Química

Avenida Randolfe Borges Júnior 1400, Univerdecidade

Uberaba - MG, CEP 38064-200, Brazil.

E-mail: kassia.santos@uftm.edu.br

Table of Contents

Page	Content
S2	Table S1. Key Contributions of Studies on Solar Energy-Based Oil Extraction Techniques.
S3	Table S2. Kinetic models of oil extraction and its applications.
S4	References

Table S1. Key Contributions of Studies on Solar Energy-Based Oil Extraction Techniques.

Reference	Key Contribution
Munir and Hensel [1]	Isolated essential oils from various peppermint species using a parabolic solar collector, with yields ranging from 4.2 to 8.2 mL kg⁻1.
Munir and Hensel [2]	Demonstrated the feasibility of integrating solar energy harvesting systems into a biorefinery concept for the extraction of high-value compounds like essential oils, phenolic compounds, and pectin, promoting sustainability and energy efficiency in the extraction process. Investigated the use of solar energy harvesting systems for peppermint oil extraction, yielding 2.00 mL/kg (Mentha piperita L.), 2.50 mL/kg (Mentha spec.), and 2.22 mL/kg (Mentha spicata).
Maiti et al. [3]	Studied the hydrodistillation of essential oil from fresh orange peels, utilizing a square parabolic trough concentrator with a 1.3 m^2 collector area. The process achieved limonene content in the crude oil ranging from 86% to 90%, with oil yields varying from 0.49% to 2.16% (w/w). The maximum hydrodistillation efficiency reached 8.37%, with oil yield closely correlating with the absorber tube temperature, which was influenced by beam radiation, ambient temperature, and wind speed.
Munir et al. [4]	Achieved efficient essential oil extraction from eucalyptus and peppermint using a solar energy harvesting system with a Scheffler reflector and steam receiver, with a break-even point of 181 days.
Shwetharani and Balakrishna [5]	Developed an environmentally friendly method for extracting bio-oil from wet algae biomass using photocatalysis, achieving a lipid yield of 52.2%.
Afzal et al. [6]	Demonstrated a low-cost and sustainable method for extracting essential oils using a solar-based hydrodistillation system, achieving extraction yields of 0.40 g/g (peppermint) and 0.59 g/g (eucalyptus) and 0.31% w/w (Pinus). Cost analysis showed a payback period of 0.5 years.
Pesimo [7]	Investigated the feasibility of using solar energy for essential oil extraction from aromatic plants, with extraction yields ranging from 18.5% (Rose petals) to 72% (Sambong) and extraction rates from 1.8 ml/day to 3.4 ml/day.
Yen and Lin [8]	Demonstrated that solar energy harvesting systems could effectively be used in hydrodistillation for essential oil extraction from herbs like clove, cumin, and fennel, with lower energy consumption compared to other herbs.
Hilali et al. [9]	Developed a zero-waste biorefinery using solar hydro-distillation to extract essential oils, polyphenols, and pectins from orange peels. Solar extraction preserved a higher amount of polyphenols and flavonoids compared to conventional methods, achieving 1 g per 100 g yield.
Radwan et al. [10]	Extracted lavender oil by varying boiler water flow rates and batch sizes, yielding 7.3 ml of high-quality lavender oil with 60.25% system efficiency and 98.13% extraction efficiency.
Ezzarrouqy et al .[11]	Investigated the efficiency of a solar hydro distillation system for rosemary leaves using a 10 m^2 Scheffler parabola, focusing on optical and thermal losses. The essential oil yield was 6.18 mL/kWh, with a total system efficiency of 40.00%, which could increase to 42.42% with steam line insulation.
Soni and Kalbande [12]	This study explores an eco-friendly method for extracting essential oil from Lemongrass using solar and biomass energy. A 16 m^2 Scheffler solar collector provided the necessary heat for hydro distillation, with biomass as a backup. The best oil yield was achieved from the 24-hour dried sample, using about 10.3 kWh of energy in the solar system. The extracted oil's properties matched those of market oils, proving the method's economic feasibility and sustainability.

Table S2. Kinetic models of oil extraction and its applications.

**Kinetic model**	**Application**	**Reference**
Esquível Model[18] $X=\frac{M_e}{M_{e0}}=\left(\frac{t}{b_1+t}\right)$ Based on experimental curve fitting, resembling the Langmuir isotherm.	Carqueja<br>shiitake<br>flaxseed<br>pink pepper<br>papaya seeds	Silva et al. [13]<br>Kitzberger et al. [14]<br>Piva et al [15]<br>Silva et al. [16]<br>Hall et al. [17]
Diffusive model[19] $X=1-A\exp(-B_1\cdot t)$ describes the process of mass transfer by diffusion according to Fick's second law, where A is the pre-exponential term, and b is the parameter related to diffusion.	sunflower oil<br>Castor bean cake<br>Canola seeds	Perez et al. [19]<br>Amarante et al.[20]<br>Fernández et al.[21]
Crank model[23] $X=\left[1-\frac{6}{\pi^2}\sum_{n-1}^{\infty}\frac{1}{n^2}\exp\left(\frac{-n^2\pi^2D_et}{r^2}\right)\right]$ Relies on Fick's second law for diffusion and assumes spherical solid particles. It considers diffusion as the primary mode of mass transfer and represents the extraction process using a diffusion coefficient and solid particle radius.	shiitake<br>pink pepper<br>rice bran oil	Kitzberger et al. [14]<br>Silva et al. [16]<br>Jesus et al. [22]
SSP model[25] $X=\left[1-\sum_{0}^{\infty}\frac{0.8}{(2n+1)^2}\exp\left(\frac{-(2n+1)^2\pi^2D_et}{\delta^2}\right)\right]$ Assumes solid particles as plate-like geometry and negligible solute concentration in the fluid phase. It accounts for mass transfer resistance in the solid phase while neglecting it in the fluid phase, assumes a plug flow pattern.	oregano bracts<br><br>marigold<br>rice bran oil	Gaspar et al. [24]<br><br>Campos et al. [25]<br>Jesus et al. [22]
Logistic Model (LM) [26] $X=\frac{1}{\exp(b\cdot t_m)}\left[\frac{1+\exp(b\cdot t_m)}{1+\exp(b\cdot(t_m-t))}-1\right]$ Based on mass balance integration and assumes constant temperature, pressure, and other fixed bed characteristics. Neglecting mass accumulation and axial dispersion.	shiitake<br>flaxseed<br>pink pepper<br>papaya seeds<br>marigold<br>rice bran oil	Kitzberger et al. [14]<br>Piva et al. [15]<br>Silva et al. [16]<br>Hall et al. [17]<br>Campos et al. 25<br>Jesus et al. [22]

X is the ratio between the oil extracted along the time

(M_e) and total initial oil content (M_{e0}); De is the diffusion coefficient, n is a integer, and r is the solid particle radius.

References

1. Munir, A., & Hensel, O. Investigation of optimal thermal parameters for essential oils extraction using laboratory and solar distillation systems. *Agricultural Engineering International: CIGR Journal*, **2010**, 12(1).

2. Munir, A., & Hensel, O. On-farm processing of medicinal and aromatic plants by solar distillation system. *Biosystems engineering*, **2010**, 106(3), 268-277.

3. Maiti, S., Bhatt, C., Patel, P., & Ghosh, P. K. Use of solar thermal energy in the hydrodistillation of essential oil. *Journal of Renewable and Sustainable Energy*, **2012**, 4(6).

4. Munir, A., Hensel, O., Scheffler, W., Hoedt, H., Amjad, W., & Ghafoor, A. Design, development and experimental results of a solar distillery for the essential oils extraction from medicinal and aromatic plants. *Solar energy*, **2014**, 108, 548-559.

5. Shwetharani, R., & Balakrishna, R. G. Efficient algal lipid extraction via photocatalysis and its conversion to biofuel. *Applied Energy*, **2016**, 168, 364-374.

6. Afzal, A., Munir, A., Ghafoor, A., & Alvarado, J. L. Development of hybrid solar distillation system for essential oil extraction. *Renewable Energy*, **2017**, 113, 22-29.

7. Pesimo, A. R. Harnessing the solar energy in extracting essential oil for community based perfumery and aromatherapy. *Open Access Library Journal*, **2017**, 4(11), 1.

8. Yen, H. Y., & Lin, Y. C. Green extraction of Cymbopogon citrus essential oil by solar energy. *Industrial Crops and Products*, **2017**, 108, 716-721.

9. Hilali, S., Fabiano-Tixier, A. S., Ruiz, K., Hejjaj, A., Ait Nouh, F., Idlimam, A., ... & Chemat, F. Green extraction of essential oils, polyphenols, and pectins from orange peel employing solar energy: toward a zero-waste biorefinery. *ACS sustainable chemistry & engineering*, **2019**, 7(13), 11815-11822.

10. Radwan, M. N., Morad, M. M., Ali, M. M., & Wasfy, K. I. A solar steam distillation system for extracting lavender volatile oil. *Energy reports*, **2020**, 6, 3080-3087.

11. Ezzarrouqy, K., Sbahi, S., Hejjaj, A., Idlimam, A., & Mandi, L. A green extraction method of phenolic compounds from olive leaves (Olea europaea L.): evaluation and prediction using multiple linear regression. *International Journal of Environmental Science and Technology*, **2024**, 21(4), 3761-3774.

12. Soni, K., & Kalbande, S. Extraction of essential oil from lemon grass through hydro distillation methods using solar and biomass energy. *International Journal of Ambient Energy*, **2024**, 45(1), 2285842.

13. Silva, D. C., Bresciani, L. F., Dalagnol, R. L., Danielski, L., Yunes, R. A., & Ferreira, S. R. Supercritical fluid extraction of carqueja (Baccharis trimera) oil: Process

parameters and composition profiles. *Food and Bioproducts Processing*, **2009**, 87(4), 317-326.

14. Kitzberger, C. S., Lomonaco, R. H., Michielin, E. M., Danielski, L., Correia, J., & Ferreira, S. R. Supercritical fluid extraction of shiitake oil: curve modeling and extract composition. *Journal of Food Engineering*, **2009**, 90(1), 35-43.

15. Piva, G. S., Weschenfelder, T. A., Franceschi, E., Cansian, R. L., Paroul, N., & Steffens, C. Extraction and modeling of flaxseed (Linnum usitatissimum) oil using subcritical propane. *Journal of Food Engineering*, **2018**, 228, 50-56.

16. Silva, B. G., do Prado, J. M., Fileti, A. M. F., Foglio, M. A., & e Rosa, P. D. T. V. Kinetic models for extraction with supercritical carbon dioxide from pink pepper: theoretical, empirical, and semi-empirical models and artificial neural network approach. *Chemical Engineering Journal Advances*, **2023**, 15, 100514.

17. Hall, R. M., Mayer, D. A., Mazzutti, S., & Ferreira, S. R. S. Simulating large scale SFE applied to recover bioactive compounds from papaya seeds. *The Journal of Supercritical Fluids*, **2018**, 140, 302-309.

18. Esquıvel, M. M., Bernardo-Gil, M. G., & King, M. B. Mathematical models for supercritical extraction of olive husk oil. *The Journal of Supercritical Fluids*, **1999**, 16(1), 43-58.

19. Perez, E. E., Carelli, A. A., & Crapiste, G. H. Temperature-dependent diffusion coefficient of oil from different sunflower seeds during extraction with hexane. *Journal of Food Engineering*, **2011**, 105(1), 180-185.

20. Amarante, R.C.A., Oliveira, P.M., Schwantes, F.K. & Morón-Villarreyes, J.A. Oil extraction from castor cake food and bioproducts processing using ethanol: kinetics and thermodynamics. *Ind. Eng. Chem.Res.*, **2016**, 53, 6824–6829.

21. Fernández, M.B., Perez, E.E., Crapiste, G.H., & Nolasco, S.M. Kinetic study of canola oil and tocopherol extraction: Parameter comparison of nonlinear models. *Journal of Food Engineering*, **2012**, 111(4), 682-689.

22. Jesus, S.P, Grimaldi, R. & Hence, H. Recovery of -oryzanol from rice bran oil byproduct using supercritical fluid extraction. *J. of Supercritical Fluids*, **2010**, 55, 149–155.

23. Crank, J. The mathematics of diffusion. Oxford university press, **1979**.

24. Gaspar, F., Lu, T., Santos, R., & Al-Duri, B. Modelling the extraction of essential oils with compressed carbon dioxide. *The Journal of Supercritical Fluids*, **2003**, 25(3), 247-260.

25. Campos, L. M., Michielin, E. M., Danielski, L., & Ferreira, S. R. Experimental data and modeling the supercritical fluid extraction of marigold (Calendula officinalis) oleoresin. *The Journal of Supercritical Fluids*, **2005**, 34(2), 163-170.

26. Martínez, J., Monteiro, A. R., Rosa, P. T., Marques, M. O., & Meireles, M. A. A. Multicomponent model to describe extraction of ginger oleoresin with supercritical carbon dioxide. *Industrial & engineering chemistry research*, **2003**, 42(5), 1057-1063.
